# The Institutional Worker

**Published:** 1912-04-06

**Authors:** 


THE
### INSTITUTIONAL WORKER
**Being a Special Supplement to "The Hospital"**

### Editor's Notices.

**Contributions:**

Contributions should be written, or preferably typed, on one side of the paper only, and all articles sent in are accepted upon the distinct understanding that they are forwarded to The Hospital only.

The Editor cannot undertake to return MSS. not used, but when a stamped directed envelope is enclosed the MSS. may be returned if a special request is made.

Accepted articles and paragraphs of news will be paid for after publication at the scale rate.

**Address:**

To prevent delay all contributions and letters on editorial business must be addressed exclusively to the Editor, "The Hospital," 29 Southampton Street, Strand, London, W.C. It is important that this regulation shall be strictly observed.

**Correspondence:**

Correspondence on all subjects is invited. The name and address of correspondents must be given as a guarantee of good faith, but not necessarily for publication.

**Special Articles:**

Special articles are invited, and questions, inquiries, and paragraphs upon all matters relating to the work, administration, and management of general, special, mental, fever, cottage and convalescent hospitals, sanatoria, homes, institutions, societies and organisations for the treatment or care of the sick, injured and dependants of all classes. Every matter affecting the interests and welfare of the staff of all grades working in these institutions will receive special consideration.

**Administrative Medicine, Research and Items of News:**

Special terms will be paid for approved articles on subjects in this wide field by experts who have made some section of it their special study and interest. We wish to give prominence to every movement tending to advance accuracy of diagnosis, efficiency in treatment, and the development of modern methods to eradicate disease and promote the welfare of the suffering.

**A Bureau of Information:**

We invite inquiries and applications for information and help in providing for that numerous class of sufferers who require special care or house-room which they cannot obtain in their own homes or secure for themselves. We cannot, however, prescribe, or recommend practitioners.

**Books for Review:**

Publishers are particularly requested to send advance proofs of any new books of importance whenever possible, as the Editor has made arrangements to publish immediate reviews on a new plan.

**Photographs, Plans, Blocks, and Illustrations:**

It is requested that wherever possible MSS. may be accompanied by illustrations in any of the above forms. The name of the sender and of the article to which it belongs should be written on the back of each photograph, drawing, or block for purposes of identification.

**Local Papers:**

Newspapers containing reports or news paragraphs should be marked and addressed to the Sub-Editor.

**The Coupon System:**

A coupon will be found at the bottom of the third inside page of the cover each week. This coupon must be attached to every question or inquiry to which an answer is desired in The Hospital.

### The Advantages of a Subscription.

The circulation of The Hospital is increasing so rapidly week by week that it is often difficult for Newsagents and Booksellers to cope with the extraordinary demand for it, especially so in some of the larger Provincial towns. Consequently, we feel it is necessary to call our readers' attention to the advantages of subscribing to the Journal either through their Bookseller or direct to the Publishing Offices. In such circumstances you are sure of securing a copy of The Hospital each week almost immediately upon publication; it reaches you regularly on a certain day and at a fixed hour; you know when to expect it, and there is no fear of your being informed that it is sold out. What a help this is when you are seeking an Appointment! as it often means that you are enabled to send in your application some hours before your competitors; this may make all the difference in securing the post, and, therefore, such an advantage is one worthy of the closest consideration. Moreover, as The Hospital is now so essential to every Institutional Worker, you cannot afford to miss even one Number, and, as a subscriber, no matter how many times you may change your address, or in what part of the country you may be at the moment, a Post Card will ensure that you receive the Journal on the same day and at the same hour as when at home.

**Subscriptions** may begin at any time, and are payable in advance. Cheques and Post-Office orders should be crossed London County and Westminster Bank, Covent Garden branch, and made payable to the Manager of The Hospital.

Rates of Subscription (payable in advance).

UNITED KINGDOM.

| | | |
|---|---|---|
| Three Months | (including Postage) ... ... ... ... | 2s. 0d. |
| Six Months | do. ... ... ... ... | 4s. 0d. |
| Twelve Months | do. ... ... ... ... | 6s. 6d. |

FOREIGN AND COLONIAL.

| | | |
|---|---|---|
| Three Months | (including Postage) ... ... ... ... | 2s. 6d. |
| Six Months | do. ... ... ... ... | 5s. 0d. |
| Twelve Months | do. ... ... ... ... | 8s. 8d. |

*SUBSCRIPTION FORM.*

*Please send me* "The Hospital" *for months,*
*commencing with your issue of*.........................., *for*
*which please find enclosed*.........................
*Name*...............................................
*Address*............................................
.........................................

Date.........................................
*To*.................................*Newsagent,*

### Manager's Notices.

**All letters relating to Subscriptions, Advertisements, and other business matters must be addressed to The Manager, "The Hospital," The Hospital Building, 28 and 29 Southampton Street, London, W.C.**

**Advertisements:**

To ensure insertion, all advertisements for The Hospital must reach the Manager not later than Wednesday morning in each week. For scale of charges see pages ix. and 2.

**Remittances:**

**It is especially requested that remittances be made by cheque or Postal Order, and in halfpenny stamps only when the amount is under sixpence.**

### Appointments Vacant.

### OFFICIAL APPOINTMENTS VACANT.

### Poor-Law Vacancies.

Assistant Cook (£24), Bermondsey Guardians. Matron of the Workhouse, Ladywell Road, Lewisham, S.E. April 25.

Assistant Laundress (£25), Lambeth Guardians. Matron of the Workhouse, Renfrew Road, Lower Kennington Lane, S.E. April 10.

Assistant Male Attendant (£25), Leicester Guardians. Mr. H. Mansfield, C. to G., Leicester. April 6.

Assistant Master (£40), York Union. Mr. G. Sykes, C. to G., York. April 9.

Assistant Nurse (£24), Maidenhead Union. Mr. W. Weed, C. to G., Maidenhead. April 9.

Assistant Nurse (£22), King's Lynn Union. Mr. R. C. Coulton, C. to G., King's Lynn. April 9.

Assistant Nurse (£20), Lambeth Guardians. Matron of the Workhouse, Renfrew Road, Lower Kennington Lane, S.E. April 10.

Assistant Nurse (£22 10s.), Hertford Union. Mr. T. J. Sworder, C. to G., Hertford. April 12.

Assistant Nurse (£22), Bridport Union. Mr. J. J. Roper, C. to G., Bridport. April 12.

Assistant Nurse (£20), Bodmin Union. Mr. J. Pethybridge, C. to G., Bodmin. April 12.

Assistant Nurse (£25), Faversham Union. G. Tassell, C. to G., Union Offices, Faversham. April 6.

Assistant Nurse (£28), Bromley Union. E. Haslehurst, C. to G., Union Offices, Park House, Bromley, Kent. April 8.

Assistant Nurse (£25), Pontypridd Union. W. Spickett, C. to G., Union Offices, Pontypridd. April 11.

Assistant Nurse (£30), Llanelly Union. D. C. Edwards, C. to G., Union Offices, Llanelly. April 8.

Assistant Nursery Attendant (£18), Lewisham Union. Mr. H. C. Mott, C. to G., 394 Lewisham High Road, S.E. April 13.

Assistant to Matron (£20), Freebridge Lynn Union. Mr. W. Cross, C. to G., King's Lynn. April 8.

Assistant Relieving Officer (£100), Mile End Guardians. Mr. B. Catmur, C. to G., Bancroft Road, Mile End, E. April 11.

Boys' Attendant (£25), Gainsborough Union. Mr. D. M. Robbs, C. to G., Gainsborough. April 8.

Boys' Charge and Recreation Master (£35), St. Pancras Guardians. Mr. J. E. P. Hall, C. to G., Town Hall, Pancras Road, N.W. April 10.

Case-Paper Clerk (£100), Kingston Union. Mr. C. W. Dash, C. to G., Kingston-on-Thames. April 8.

Charge Nurse (£30), Dudley Union. Mr. E. Allen, C. to G., Dudley. April 10.

Charge Nurse (£35), Bromley Union. E. Haslehurst, C. to G., Union Offices, Park House, Bromley, Kent. April 15.

Charge Nurse (£30), West Ham Union. Mr. T. Smith, C. to G., Leytonstone, N.E. April 10.

Charge Nurse (£32), Huddersfield Union. E. A. Rigby, C. to G., Union Offices, Ramsden Street, Huddersfield. April 11.

Charge Nurse (£30), West Ham Union. T. Smith, C. to G., Union Road, Leytonstone, N.E. April 10.

Engineer (35s. weekly), Bermondsey Guardians. Mr. E. Pitts Fenton, C. to G., 283 Tooley Street, S.E. April 24.

Female Attendant (£25), Reading Guardians. Mr. W. H. Oliver, C. to G., Reading. April 11.

Female Assistant Relieving Officer (£75), Chesterfield Union. R. F. Hartwright, C. to G., Union Offices, Chesterfield. April 12.

Female Cook (£35), Merthyr Tydfil Union. Mr. F. T. James, C. to G., Merthyr Tydfil. April 11.

Female Lunatic Attendant (£18), Cardiff Union. Mr. A. J. Harris, C. to G., Cardiff. April 18.

First Assistant Master's Clerk (£30), Willesden Guardians. Mr. J. H. Haylor, C. to G., 357 High Road, Brondesbury, N.W. April 9.

Foster-Mother (£22), Newton Abbot Union. Mr. F. Horner, C. to G., Newton Abbot. April 9.

Foster-Mother (£25), Guildford Union. Mr. W. S. V. Cullerne, C. to G., Guildford. April 12.

Girls' Industrial Trainer (£25), Gainsborough Union. Mr. D. M. Robbs, C. to G., Gainsborough. April 8.

Head Nurse (£32), Camberwell Guardians. Mr. C. S. Stevens, C. to G., Peckham Road, S.E. April 17.

Laundress (£30), Woolwich Union. Mr. T. Cutter, C. to G., Plumstead. April 8.

Laundry Superintendent (£30), Bristol Guardians. Mr. J. J. Simpson, C. to G., Bristol. April 11.

Male Night Nurse (27s. weekly, non-resident), Cardiff Union. Mr. A. J. Harris, C. to G., Cardiff. April 18.

Master's Clerk (22s. weekly), North Bierley Union. Mr. W. G. Cooper, C. to G., 4 Town Hall Street, Bradford. April 8.

Matron's Assistant (£25), Glanford Brigg Union. Mr. F. C. Hett, C. to G., Brigg. April 10.

Night Nurse (£27 10s.), Battle Union. Mr. F. G. Ticehurst, C. to G., Battle. April 18.

Nurse (£30), Wantage Union. Mr. E. B. Ormond, C. to G., Wantage. April 6.

Nurse (£30), Wigan Union. Mr. H. G. Ackerley, C. to G., Wigan. April 9.

Nurse (£30), Barnet Union. Master of the Workhouse, Barnet. April 10.

Nurse (£30), Abergavenny Union. Mr. W. H. P. Scanlon, C. to G., Abergavenny. April 17.

Nurse (£40), Plympton St. Mary Union. Mr. J. W. Bickle, C. to G., Westwell Street, Plymouth. April 11.

Nurse (£25), Worksop Union. J. S. Whall, 66 Bridge Street, Worksop. April 9.

Old Women's Attendant (£20), Holborn Union. Matron of the Workhouse, 1a Shepherdess Walk, N.

Probationer Nurses (£10), Eastbourne Union. A. Hurst, C. to G., Avenue Home, Eastbourne.

Recreation Mistress (£30), St. Pancras Guardians. Mr. J. E. P. Hall, C. to G., Town Hall, Pancras Road, N.W. April 10.

Receiving Ward Nurse (£28), Holborn Union. Matron of the Workhouse, 1a Shepherdess Walk, N.

Relieving Officers (2) (£100 each), Walsall Union. Mr. A. H. Lewis, C. to G., Walsall. April 11.

Relieving Officer, etc., Pontypool Union. Mr. T. Watkins, C. to G., Pontypool. April 16.

Staff Nurse (£22), Camberwell Guardians. Mr. C. S. Stevens, C. to G., Peckham Road, S.E. April 18.

Stores Porter (£25), Leicester Guardians. Mr. H. Mansfield, C. to G., Leicester. April 6.

Superintendent Nurse (£60), Coventry Union. Mr. E. A. Evans, C. to G., Coventry. April 13.

Wardmaids (£17), Guildford Union. Mr. W. S. V. Cullerne, C. to G., Guildford. April 12.

### Institutional and Administrative Vacancies.

Housekeeper (£50), Prince of Wales's General Hospital, Tottenham. The Chairman of the House Committee.

Matron (£60), Royal Victoria Hospital, Bournemouth. Gordon Martyr, Secretary. April 8.

Matron (£45), Ilkeston Hospital. A. J. Worrall, Secretary, 18 Drummond Road, Ilkeston. April 17.

### Municipal Vacancies.

Assistant Land Agent (£120), Dorset C.C. Mr. E. A. Ffooks, Clerk to the Council, Dorchester. April 10.

Building Inspector (£100), Plymouth T.C. Mr. J. Paton, Borough Surveyor, Plymouth. April 10.

Clerk of Works (Street Improvements) (50s. weekly), Hale U.D.C. Mr. J. G. Whyatt, Clerk to the Council, Hale, Cheshire. April 11.

Draughtsman (£150), Bolton T.C. Mr. S. Parker, Town Clerk, Bolton. April 10.

Female Dispenser (£100), M.A.B. Clerk to the Board, Embankment, E.C. April 10.

Female Sanitary Inspector (30s. weekly), Bury T.C. Mr. J. Haslam, Town Clerk, Bury. April 9.

Head Constable (£450), Newport T.C. Mr. A. A. Newman, Town Clerk, Newport, Mon. April 10.

Highway Surveyor and Inspector of Nuisances (£180), Claypole R.D.C. Mr. A. J. Franks, Clerk to the Council, Newark. April 8.

Highway Surveyor (£120), Totnes R.D.C. Mr. F. K. Windeatt, Clerk to the Council, Totnes. April 10.

Inspector of Nuisances (£100), Coseley U.D.C. Mr. W. Lees, Clerk to the Council, Coseley. April 6.

Inspector of Nuisances (£104), Kiveton Park R.D.C. Mr. J. S. Whall, Clerk to the Council, 66 Bridge Street, Worksop. April 8.

Inspector of Weights and Measures (£175), East Sussex C.C. Mr. F. Merrifield, Clerk to the Council, Lewes. April 19.

Midwifery Nurse (£100), Manchester T.C. Mr. T. Hudson, Town Clerk, Manchester. April 13.

Nurse-Matron (£40), Maidstone Borough Sanatorium. S. L. Monckton, Town Clerk, Maidstone. May 7.

Sewage Farm Sub-Manager (£100), Coventry T.C. Mr. G. Sutton, Town Clerk, Coventry. April 15.

Superintendent Nurse (£42), M.A.B. The Clerk to the Board, Embankment, E.C. April 16.

### Miscellaneous Vacancies.

Instructor in Shoemaking and Discipline Master for Industrial School (£40), L.C.C. Education Offices, Victoria Embankment, W.C. April 6.

School Nurse (£75), Essex Education Committee. Mr. J. H. Nicholas, Secretary to the Committee, Chelmsford. April 11.

School Nurse (£80), Glamorgan County Council. Dr. D. J. Morgan, 10 The Parade, Cardiff. April 12.

